# Strengthening digital monitoring of antibiotic resistance in low-resource settings

**DOI:** 10.7189/jogh.12.03061

**Published:** 2022-10-01

**Authors:** Yogita Thakral, Sundeep Sahay, Arunima Mukherjee

**Affiliations:** 1 HISP Centre and Department of Informatics, University of Oslo, Norway; 2 HISP India, New Delhi, India

Antimicrobial resistance (AMR) is a tremendous contemporary challenge, described by the former secretary-general of the World Health Organization (WHO), M Chan as a “slow-moving tsunami” threatening “the end of modern medicine as we know it” [1]. AMR has multi-faceted adverse consequences spanning human and animal health, economics, climate, and environment, endangering the future of societies at large and the achievement of nearly all the SDGs [2]. Since the late nineties, various global declarations and research publications have emphasized the need to combat AMR, such as the 1998 World Health Assembly (WHA) Resolution and 2001 WHO Global Strategy for Containment of Antimicrobial Resistance. In May 2015, the 68th WHA adopted the global action plan on AMR, urging all member states to implement National Action Plans (NAPs) by 2017; while many countries helped in formulating these plans, they lag far behind in their implementation, particularly low- and middle-income countries (LMICs). Antibiotics resistance (ABR) is a subset of AMR which specifies resistance in bacteria associated with antibiotics. This paper focuses on the challenge of implementing a key recommendation of the NAP, which is to strengthen the knowledge and evidence base through surveillance and research. LMICs face the dual problem of high ABR burdens and weak monitoring systems [2] leading to a vicious cycle of inadequate knowledge of ABR and interventions designed without adequate scientific evidence base leading to further ABR spread. A striking example of this is the rising misuse of antibiotics. In India, a global ABR hotspot which continues to show very high levels of consumption of antibiotics, this is reflected in an increase of more than 100% over the period 2000-2015 [3]. Lack of systematic monitoring contributes to this significant rise with consequences on ABR prevalence.

Information and communications technology (ICTs) can play a key role in monitoring for making improvements at both policy and clinical levels. For policy, monitoring can help in understanding where and what ABR spread, which can guide policies on resource allocation and regulatory frameworks. Clinically, effective monitoring supports targeted treatment and helps with strengthening infection control practices and developing guidelines for antibiotic prescription practices. Science has emphatically argued for the need to strengthen monitoring systems in combating ABR, but how this can be done in practice remains both a research and practical challenge.

ABR represents a unique challenge in two dimensions due to its scale and scope. First is the geographical dimension, since ABR represents a national and global problem without any geographical constraints. Second is the functional dimension, since ABR monitoring is grounded within the One Health (OH) approach which acknowledges the interconnectedness of humans, animals, and the environment [4]. An ABR monitoring platform for the human domain needs to be scalable to other domains of veterinary medicine and the environment, to better understand the source and transmission of infections.

## WHAT ARE APPROACHES TO DESIGNING ABR MONITORING SYSTEMS RELEVANT FOR ADDRESSING MULTIPLE GEOGRAPHICAL CONTEXTS AND VARYING FUNCTIONAL REQUIREMENTS IN RESOURCE-CONSTRAINED SETTINGS?

As one of the largest contemporary global health threats, ABR is estimated to contribute annually to about 700 000 deaths, rising to an estimated 10 million deaths and a cost of US$100 trillion by 2050 [5]. The threat is aggravated by a 65% global increase in human antibiotic consumption during 2000-2015 and an 80% rise in the use of antibiotics in the animal sector [3]. While India is considered a global ABR hotspot, the magnitude of the problem is largely unknown because of weak monitoring systems, which has global consequences with the intensification of globalization processes exemplified by movements of more than 1 billion people across borders annually [6], including tourism to the tropics often colonized by resistant microbes [7]. Most LMICs have weak monitoring systems [8], making their strengthening an urgent priority. In 2014, five of the 11 Southeast Asian Region countries (India, Bangladesh, Indonesia, Maldives, and East Timor) could not generate systematic ABR data. Only Thailand and Nepal showed the capacity to report data from more than five laboratory sites [9]. As a result, governments’ possibilities to establish the epidemiological links between rampant use of antibiotics and ABR and determine how spread across geographical boundaries and functional domains (humans, animals, environment, and food) are limited. While the importance of monitoring in the fight against ABR is universally acknowledged, methods of execution are discussed less, which is particularly relevant for low-resourced settings, across multiple facility types and use cases.

Our work is based in India, where the challenge of scale is particularly acute, given the high numbers of health facilities, population, and levels of ABR prevalence. Many millions of people, particularly in rural areas, lack access to relevant drugs and diagnostic facilities (typically available only in capital cities) and face financial constraints [4,9]. Samples from district hospitals (more than 800 in India) need to be sent to the tertiary hospitals, representing a huge logistics challenge. India also suffers from drastic misuse and overuse of antibiotics, as it flourishes in the absence of regulatory frameworks and antibiotics usage guidelines[10]. Data reporting from private facilities to national systems is minimal, even though they are the biggest source of laboratory testing, and almost nothing is known about ABR prevalence in agriculture, environment, and veterinary domains [1]. These conditions make designing an ABR monitoring system relevant for multiple settings and used in low-resource settings a significant challenge. In this paper, we discuss an empirical engagement of designing and implementing an ABR monitoring system for a public facility in India and making it relevant for multiple other settings. We are guided by information infrastructure theory to address this design challenge.

### Conceptualizing the design challenge: an information infrastructure (II) perspective

Information infrastructure (II) theory helps understand the design and evolution strategies of large-scale, complex [11], and distributed systems like the Internet [12] and national health monitoring systems. IIs represent interconnected technical and institutional elements, without finite start and end dates, which are forever evolving. IIs are shared, and no one single entity controls the whole infrastructure. The nature of heterogenous interconnections and their dynamic nature make them complex and tackled differently from traditional stand-alone systems [11,12]. IIs involve multiple and heterogeneous stakeholders with asymmetric power relations and conflicting goals, requiring diverse, dynamic, and novel design approaches [13,14].

An ABR monitoring platform within an OH framework is best conceptualized as an II, as it requires managing data from multiple domains with different subsystems through technical and institutional collaborations. Within a domain are multiple sub-systems of sample collection, laboratory testing, patient clinical conditions and treatment, and dissemination of results for policy and practice improvements. Expanding this to the OH, where there are multiple domains involved (human, animals, environment, food), owned by different entities (ministries of health and animal husbandry), the complexity of monitoring expands manifold. II theory emphasizes the need for cross-boundary and disciplinary knowledge to “force unity from diversity, centralization in the face of pluralism, and coherence from chaos” [15].

**Figure:**
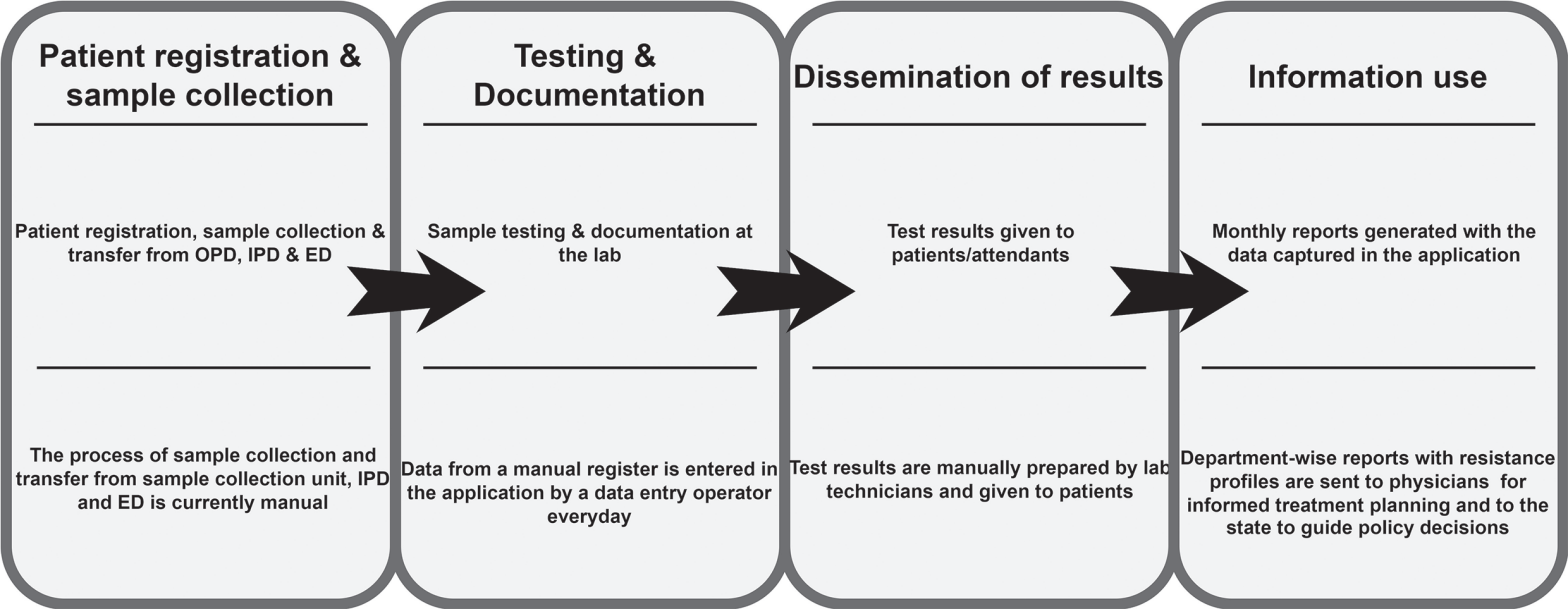
Photo: The information flow digitised at the microbiology testing laboratory. Source: original picture.

Two key design challenges identified by II theory concern bootstrapping and adaptability. Bootstrapping refers to the early phase of an II evolution when there are limited users, thus providing limited value in attracting new users, which constrains growth. The implication of this for ABR monitoring is that bootstraping by attracting new users becomes hard in early stages where such systems are largely non-existent. Adaptability concerns the challenge of making well-entrenched systems compatible with new environments. As the ABR monitoring system expands and takes root, incorporating new informational requirements may become difficult, which is inherently the case in this evolving domain.

To deal with these two problems, Hanseth and Lyytinen [11] proposed five design principles (DPs) which serve as broad guidelines to help “formulate in concrete terms how to generate and select desired system features as to achieve stated system goals”. DP1 is designed for direct usefulness by offering relevant functionalities for a small group, not requiring dealing with the bootstrapping problem. DP2 is made to build upon the existing systems by incorporating new functionalities to create added value for user groups. DP3 concerns gathering use momentum by expanding the systems and through the use of persuasive tactics to enroll new uses and users. DP4 makes the II as simple as possible for users, and DP5 is supposed to modularize the systems by minimizing tightly coupled dependencies and buffer to minimize risks of full breakdowns when one part of the II malfunctions. We use these DPs as our initial guidelines, to design a monitoring application for one setting and then slowly build upon that to make it relevant for multiple settings in low-resourced contexts.

#### ABR monitoring system design

The system is built upon the free and open-source District Health Information System (DHIS2) platform endorsed by many global partners such as WHO (see www.dhis2.org) and applied for supporting multiple types of applications in national health systems. We describe the processes of its building and expansion at the five levels described above.

Level 1 – regional: A national-level research organization (anonymized as InMo) had, since 2016, established an ABR surveillance network comprising 27 specialty hospitals and private sector laboratories across India. The platform was built in-house and was experiencing functional scaling challenges, particularly related to analytics. Since InMo was already using DHIS2 internally for other applications, they decided to replicate the existing ABR platform on DHIS2 and approached a local non-governmental organization (NGO) called HIndia (HI) to undertake this project, as they had long-standing DHIS2 expertise and agreed to do the development without cost.

HI took an incremental design approach by first replicating the existing data entry module in DHIS2, followed by the output module. Requirements were understood by studying existing design documents, discussing with InMo developers, and seeing system demos. Initial requirements were shared by InMo in the form of Excel sheets extracted from the application’s database and used to build the new design blueprint. The design process was unsatisfactory, as HI was not permitted to meet the end-users and requirements were only narrated through the InMO team. Many requirements were thus lost in translation, leading to many rounds of fieldwork, causing frustration to HI. After more than a year of work, as HI saw the application not being put to real use, they discontinued the project.

Level 2 – The health facility: A positive outcome of the InMo work was that the HI team got exposed to the ABR domain and the terminology that a monitoring application entailed. To build on this learning, they approached a public tertiary hospital facility (anonymized as THospital (TH)) in a northern state (anonymized as NState) to build a monitoring platform for the facility, particularly to support the sample testing process in the microbiology laboratory. The initial design was based on the user requirements at the microbiology laboratory through discussions with the laboratory staff, who could explain their workflow, which they wanted to be digitally replicated. The HI team took as the reference the DHIS2 application they had built for InMO and started a process of concretely asking the users what was different from what they needed. The users felt that the application was for reporting monitoring data to the regional level, primarily to support research, it was not appropriate for micro-level laboratory work. The HI team started a design process from scratch, spending time to understand the information flow relating to testing samples reaching the laboratory, the sample details being entered in manual registers, followed by the test results (whether the sample was positive or not), and the creation of different outputs showing patterns of resistance.

Using a prototyping approach, the developers created the functionalities requested by users through different iterative cycles (requesting user feedback, incorporating required changes, and releasing them for use). After more than 18 months of such iterative cycles, the application has stabilized and more than 200 test records are entered into the system as of now, with more than 12000 records in the database.

Level 3 – intra-facility: After the digitization process at the microbiology laboratory was stabilized, HI started work on improving data quality and improving information use. To improve data quality, HI hired a full-time “data officer” who systematically studied the indent forms received at the laboratory from the hospital departments. Many missing data fields were identified, such as the details of the antibiotics the patient had been prescribed. Missing data reports were given to the laboratory team who acknowledged this limitation and started to contact the indenting doctor to provide more complete details. Gradually, the quality of data are being improved, along with trust in it to circulate for inter-departmental use.

To enhance information use, the HI team worked with the laboratory staff to understand how data would like to be used, both in the laboratory and in other departments. The laboratory staff started giving requirements for different kinds of output reports, to which the HI team continuously responded. For the first time, the staff could now see trends in the resistance data through attractive visualizations made available through the dashboard.

A key limitation was that these outputs being generated were not being put to broader use in the health facility, particularly by the treating doctor and the Hospital Infection Control Committee. The HI team then made many new outputs; for example, department-wise reports to guide the physician in prescribing evidence-based antibiotics and the hospital administration in making antibiotics policy. Further, this required a change from organism to patient sample specific.

The HI Data Officer is now also engaged in tasks beyond data entry, by taking the output reports in printout form (as most departments do not have computers and data can’t be shared digitally) to clinicians and hospital administrators and explaining how the reports can be understood and used.

Level 4 – interfacility, within and across states: Since TD was keen to shasre their learning and experience with other nearby hospitals, which had the required microbiology testing facilities, their principal called for a workshop in July 2021, which was attended by staff from the other hospitals. The TD microbiology team very proudly made a presentation of their achievements, which generated great interest among the other hospitals, believing that “if our TD could do it, then so can we”. At least two of the nearby hospitals have now agreed to adopt similar systems and processes. The HI data officer is facilitating this adoption process and these two hospitals are expected to have operating systems in about three months. The implementation cycle is significantly compressed, as HI can now leverage the experience and use the already-developed platform without having to reinvent the wheel. An important implication of this effort, when successful, would be that the state administrators could get a relatively robust picture of the ABR trends, which could support evidence-based policy interventions.

Through their existing networks, HI was approached by another (called BState) to strengthen monitoring in ten medical colleges in their state. Since BState was quite different from NState on many fronts (it had a poorer infrastructure, higher population, more reported antibiotics consumption, and higher rates of infection), it was belived that a similar process of expansion may not work in BState. A decision was made to create a team comprising of local medical staff, a HI staff member, and two national ABR experts to survey the microbiology testing infrastructure in the ten facilities. The idea was to assess what digital infrastructure existed for supporting HR capacity and what tests were being done. Through this survey, three hospitals have been identified to initiate the (re)design and implementation of the monitoring application.

Level 5 – global: The DHIS2 is a globally accepted platform and is currently in use in more than 80 countries for the development of different kinds of health information applications. The University of Oslo (UiO), from where the development of DHIS2 is coordinated, is a WHO Collaborating Centre and had released many of its health program-specific apps (such as for HIV and TB) on the DHIS2 platform. Seeing the potential of HI’s work in India, the WHO is keen to explore similar possibilities for the ABR application to be shared by them as a global good. HI and WHO have been discussing this for more than a year and HI has worked to improve their application’s global applicability by integrating data sharing mechanisms with two WHO ABR applications: WHONET [16] and the Global Antimicrobial Resistance Surveillance System (GLASS). Since the WHONET is widely used in laboratories globally, integration with it helps to build on an existing installed base. Since GLASS is a WHO-mandated system for national ABR reporting, integration will help India with reporting annual ABR statistics to WHO. Further, HI has also integrated the system with ICD11 (International Classification of Diseases) standards to enhance global compatibility. Convinced of the HI application’s value, there is a plan to pilot its implementation in Laos in 2022.

Another ongoing expansion process is through the contracting of HI by a German research consortium to implement the application in six hospitals across five African countries. Conducted online during COVID-19, the HI demonstrated their India application to different consortium partners who used it as a reference to specify their additional requirements. After more than a year of interactions, the requirements have been finalized for the different hospitals, particular applications configured, and three of the six hospitals have installed it and started data entry. The other three are expected to go live in the next three months.

#### Making design relevant for multiple settings

Guided by II design principles, HI has been engaged in a long-term process of making the ABR monitoring application relevant for multiple settings, all within low-resource contexts.

DP1 – design for direct usefulness: This DP was difficult to apply in the regional level system, as it was difficult to determine useful features without direct access to users. At the facility level, HI understood specific requirements which would benefit the laboratory (digitization of testing process) and other departments (department-wise ABR reports) and could rapidly provide for the required functionalities. At BState, the assessment of the laboratories provided a basis for understanding what would be useful for WHO; a key value-adding feature was the integration of the application with the 2 global WHO systems. For the German consortium hospitals, detailed engagement with users for requirements has helped to identify use adding features, which have gradually been incorporated into the application.

DP2 – build upon the existing installed base: II theory informs that the existing installed base has both enabling and constraining influences. At the regional level, the existing regional system which HI was mandated to replicate, represented the installed base, which severely constrained the development process. At TD, the absence of a digital system served as virgin grounds to positively experiment with digitization, which would also be the case in BState hospitals. At WHO, their existing systems (WHONET and GLASS) provide an extensive installed base, and enabling data sharing of the HI application with them could help HI leverage on existing systems and the WHO legitimacy.

DP3 – expand installed base by persuasive tactics to enroll new users: Existing champions who could persuade others to adopt the systems play a key role. At the regional level, no such champion emerged, while at TD, the microbiology laboratory users championed the adoption of the system and its outputs, both within and across their facilities. The leaders of the German consortium championed the use of the system in the six facilities, while the WHO HQ ABR team is spearheading efforts at the global level. The assessment exercise carried out in BState through an expert multi-disciplinary team provides for the legitimacy of the arguments to strengthen the monitoring systems.

DP4 – make the II as simple as possible: The design process was both constrained and enabled by the DHIS2 core architecture. At the regional level, since the development task was replication rather than designing from scratch, HI could not consciously design for simplicity. At the facility level and in subsequent developments, simplicity has always been a guiding principle – for example, through replicating existing forms and workflows of the laboratory technicians to promote familiarity. The approach has always been one of incremental development, where small changes are made in quick iterative cycles, with feedback elicited and built upon.

DP5 – modularize the II: The DHIS2 has a modular structure (see technical documentation at www.dhis2.org) by design, including multiple apps for data entry, outputs, transfer isolation, WHONET and GLASS integration, and data sharing with third-party apps. As new requirements are identified, new modules/functionalities can be added on. New users have the option to use all or some of these apps for their purposes. We summarise the different modes through which the multi-site expansion of the ABR monitoring application has taken place since 2018 in **Table 1**.

**Table 1:** Modes of expansion

Levels of expansion	Mechanism of scaling	Status
Facility	Used regional-level application as a baseline to build for facility-level application.	Application in use at the facility since 2019; ongoing enhancements implemented based on requirements.
Intra-facility	Improving data quality, department-wise reports.	Data quality analyst hired to improve both manual and digital data quality; department-wise reports developed and shared with other departments.
Inter-facility and within other states	Interfacility: Learnings from the existing hospital to be taken to new hospitals; Within other states Assessment of microbiology laboratories for capacity, logistics, and infrastructure for conducting tests and using the application.	Interfacility: On-going discussions on capacity and infrastructure to start using the application; Within other states: A preliminary assessment was conducted; pilot sites identified.
Global	Existing application as the baseline application to configure monitoring application for six sub-Saharan African countries; WHONET and GLASS integration.	The pilot started in three countries: ongoing discussions with two; ongoing discussions with WHO HQ.

ABR is a global problem, with interconnections between different geographies and functional domains. Clinicians and laboratory staff at the facility level and policymakers at state, regional, and global levels require different kinds of information to support different purposes of clinical practice, policy, and research. The ABR application thus needs to be made relevant for different settings and use cases.
